# Low-intensity resistance exercise with blood flow restriction for patients with claudication: A randomized controlled feasibility trial

**DOI:** 10.1177/1358863X231200250

**Published:** 2023-10-11

**Authors:** Thomas Parkington, David Broom, Thomas Maden-Wilkinson, Shah Nawaz, Markos Klonizakis

**Affiliations:** 1 Department of Nursing and Midwifery, Lifestyle, Exercise and Nutrition Improvement Research Group, Sheffield Hallam University, Sheffield, UK; 2 Department of Sport and Physical Activity, Physical Activity, Wellness and Public Health Research Group, Sheffield Hallam University, Sheffield, UK; 3 Centre for Sport, Exercise and Life Sciences, Coventry University, Coventry, UK; 4 Sheffield Vascular Institute, Sheffield Teaching Hospitals NHS Foundation Trust, Sheffield, UK

**Keywords:** exercise performance, peripheral artery disease (PAD), quality of life, rehabilitation, strength training

## Abstract

**Background:**

Claudication is a common and debilitating symptom of peripheral artery disease, resulting in poor exercise performance and quality of life (QoL). Supervised exercise programs are an effective rehabilitation for patients with claudication, but they are poorly adhered to, in part due to the high pain and effort associated with walking, aerobic, and resistance exercise. Low-intensity resistance exercise with blood flow restriction (BFR) represents an alternative exercise method for individuals who are intolerant to high-intensity protocols. The aim of this study was to evaluate the feasibility of a supervised BFR program in patients with claudication.

**Methods:**

Thirty patients with stable claudication completed an 8-week supervised exercise program and were randomized to either BFR (*n* = 15) or a control of matched exercise without BFR (control; *n* = 15). Feasibility, safety, and efficacy were assessed.

**Results:**

All success criteria of the feasibility trial were met. Exercise adherence was high (BFR = 78.3%, control = 83.8%), loss to follow up was 10%, and there were no adverse events. Clinical improvement in walking was achieved in 86% of patients in the BFR group but in only 46% of patients in the control group. Time to claudication pain during walking increased by 35% for BFR but was unchanged for the control. QoL for the BFR group showed improved mobility, ability to do usual activities, pain, depression, and overall health at follow up.

**Conclusion:**

A supervised blood flow restriction program is feasible in patients with claudication and has the potential to increase exercise performance, reduce pain, and improve QoL. (**Clinicaltrials.gov Identifier: NCT04890275**)

## Background

Supervised exercise programs are effective rehabilitation for patients with peripheral artery disease (PAD)-related claudication, delivering improvement in exercise performance, quality of life (QoL), and exertional leg symptoms.^1^ This is important as symptoms usually worsen with time.^2^ Guidelines for these programs recommend interval walking as the primary mode of exercise in addition to other modes of aerobic exercise (e.g., upper body ergometry or cycling) and resistance exercise.^3^ However, uptake and adherence to supervised exercise programs are poor,^4^ in part due to high pain and the effort associated with exercise, which presents a substantial motivational challenge for patients.^5^ Therefore, there is a need to present alternative exercise modes.

Low-intensity resistance exercise with blood flow restriction (BFR) is becoming popular as a rehabilitation tool for clinical populations that are intolerant to high-intensity protocols.^6^ The BFR technique involves a pneumatic cuff on the proximal aspect of the exercising limb to apply a pressure sufficient to occlude venous flow while lifting low loads (20–40% 1RM (one-repetition maximum); 15–30 repetitions per set).^7^ The acute response to the application of the cuff is turbulent artery blood flow, reduced intramuscular oxygen delivery, decreased venous clearance of metabolites, and blood pooling within the capillaries.^8^ This response elevates levels of metabolic stress during exercise, which causes an increase in muscle fiber recruitment and accelerates the onset of peripheral fatigue.^9,10^ When the cuff is released, reperfusion and shear stress initiate a vasodilatory and/or enhance blood flow response.^11^

Muscle perturbations and hemodynamic disruption facilitated by the cuff are thought to activate systemic hormone production,^12^ myofibrillar and mitochondrial protein synthesis,^13,14^ angiogenesis,^15^ and mitochondrial biogenesis.^16^ As a result, a program of BFR training induces hypertrophy, strength, and muscular endurance comparable to high-intensity resistance training, despite using low workloads.^17–19^ Therefore, BFR may be useful for clinical populations when high mechanical stress and psychological challenge associated with exercise performed at high intensity is contraindicated or unfeasible. Many previous studies have shown BFR to be safe and effective in varying clinical populations.^20–25^ Additionally, BFR has been shown to improve physical function, including walking performance, in healthy sedentary older adults,^26^ sarcopenic women,^27^ and patients with heart failure.^28^

BFR represents an alternative exercise method for aiding rehabilitation and has potential utility in patients with claudication. Although BFR protocols appear safe and acceptable to a variety of populations,^20–25,29^ the use of BFR with claudication patients has not been previously investigated; therefore, the possibility of unfavorable effects cannot be excluded. The aim of this study was to evaluate the feasibility of a supervised BFR program in a small claudication patient cohort. Such a preliminary study is important prior to the evaluation of the clinical and cost-effectiveness of BFR in a large patient cohort.^30^

## Methods

The study was a two-arm, parallel group, randomized controlled feasibility trial conducted in Sheffield (UK), which was developed and delivered as current standard practice.^31^ Ethics approval was granted by the NHS National Research Ethics Service, Yorkshire and the Humber (Leeds) Committee (20/YH/0039), with the study conducted in accordance with The Code of Ethics of the World Medical Association (Declaration of Helsinki). The study was prospectively registered as Clinicaltrials.gov Identifier: NCT04890275.

Patients were recruited from the claudication clinics by Sheffield Vascular Institute of Sheffield Teaching Hospitals NHS Foundation Trust.

Eligibility criteria included: (1) diagnosed PAD with stable claudication (i.e., symptomatic presentation unchanged for 6 months); and (2) ankle–brachial index (ABI) ⩽ 0.9. Exclusion criteria included: (1) stents in the artery system of the thigh; (2) ABI > 0.89; (3) symptomatic presentation of rest pain, skin ulcers, or gangrene; and (4) impaired walking by a non-PAD condition (e.g., osteoarthritis of hip or knee joint) or cannot walk without a walking aid.

All patients provided written informed consent. As this was a feasibility study, no formal sample size calculation was required. The aim was a sample of 30 patients, which is suitable for a feasibility trial to provide sufficient precision of the mean and variance.^32^

### Randomization and allocation

Following baseline assessments, patients were randomly assigned 1:1 to the experimental exercise group (BFR) or the active comparator group (control). Patients were stratified by ABI (⩾ 0.7 and < 0.7) and sex.

### Supervised exercise programs

Patients in both the BFR and the control groups received supervised exercise sessions at the Exercise Research Laboratory (Centre of Sport and Exercise Science, Sheffield Hallam University), twice weekly for 8 weeks (total of 16 sessions), directed by an experienced exercise physiologist (TP). This frequency with progressive overload is sufficient to stimulate hypertrophy and strength^33^ with adaptations observed from 4 weeks that may lead to greater muscular improvements with longer durations.^34,35^

All sessions began with a warm-up of 5 minutes of light cycling. Patients controlled the cadence and load which corresponded to a rating of perceived exertion (RPE) of 9–11 on the category-ratio (CR-20) scale.^36^ Following this, the main part of the session consisted of lower-body resistance exercises: leg press (Pro2 Seated Leg Press; Life Fitness, Chicago, IL, USA) and knee extension (SP100; TECA Fitness, Montesilvano, Italy). Patients performed four sets of 30, 15, 15, 15 repetitions of leg press followed by three sets of 15, 15, 15 repetitions of knee extension at 20% 1RM. Exercises were performed bilaterally with repetitions executed every 3 seconds (1.5 s during the concentric phase and 1.5 s during the eccentric phase) with support from a metronome. Exercise for BFR and control groups was matched at a relative volume-load.

**Figure 1.**
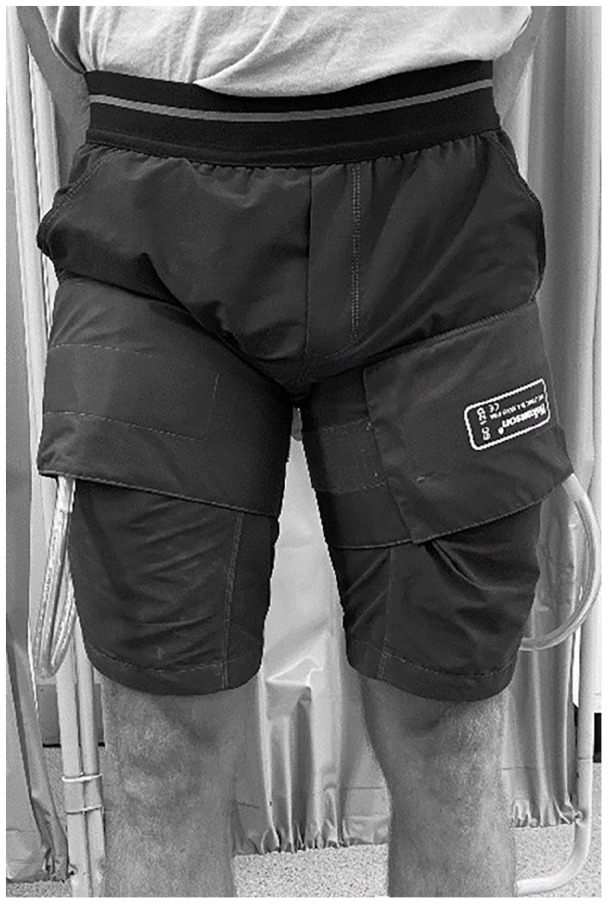
Placement of the pneumatic cuff on the legs.

Patients in the BFR group completed the resistance exercises with the addition of a pneumatic cuff (13 cm wide, SC12L segmental pressure cuff, E20 Rapid Cuff Inflator, and AG101 Cuff Inflator Air Source; Hokanson, Indianapolis, IN, USA) placed around the proximal aspect of the legs (Figure 1). The pneumatic cuff was inflated 10 seconds before starting each resistance exercise, remained inflated during exercise, including the in between sets rest period, and was deflated immediately after exercise completion. The pneumatic cuff pressure was set to 50% arterial occlusion pressure (149.6 ± 35.4 mmHg) in accordance with guidelines.^7^

Arterial occlusion pressure was assessed at baseline following established procedures.^37^ The lowest arterial occlusion pressure of the legs was used to set the pneumatic cuff pressure. Typically, the lowest arterial occlusion pressure was recorded in the leg most affected by PAD. If complete arterial occlusion could not be achieved by 220 mmHg, the pressure recorded was capped at 220 mmHg to minimize undue pain for the patient. This occurred in two patients.

**Figure 2.**
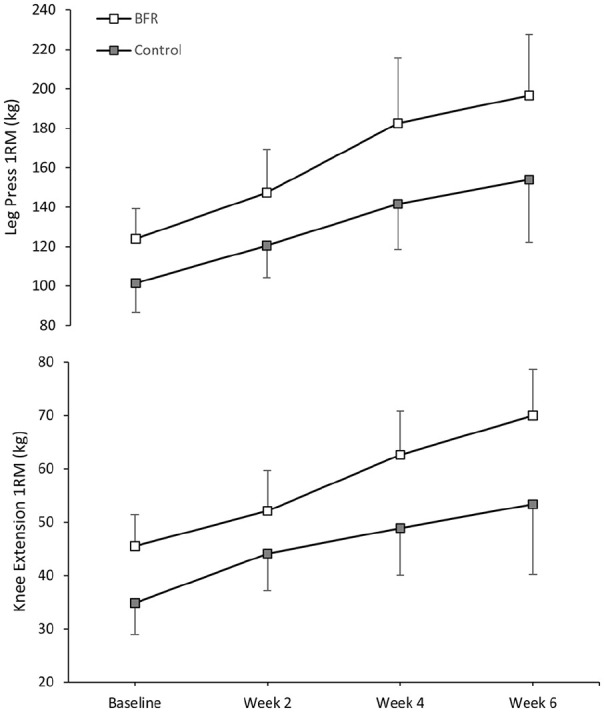
Predicted one-repetition maximum through the supervised exercise program illustrating progressive overload. Data are mean ± standard error of the mean.

Leg press and knee extension 1RM was predicted, using the repetitions to failure method as previously described,^29^ at baseline to determine the load used during the resistance exercises. For progressive overload, predicted 1RM was retested every 2 weeks to recalculate the load throughout the 8-week program (Figure 2). Both groups observed an improvement in 1RM from baseline to week 6 for leg press (BFR = 72.6 kg [31.1, 114.1], control = 52.4 kg [11.4, 93.3]) and knee extension (BFR = 24.5 kg [11.4, 37.6], control = 18.6 kg [5.1, 32.2]).

### Procedures

During Visit 1, after written informed consent had been obtained and eligibility confirmed (determined by the study physician SN), the following baseline measurements were recorded: (1) demographic data; (2) height and body mass; (3) ABI, assessed via the Doppler ultrasound technique;^38^ (4) vastus lateralis muscle thickness via B-mode ultrasonography (Sonimage MX1; Konica Minolta, Tokyo, Japan) following standardized procedures;^39^ (5) QoL questionnaire (ED-5D-5L); (6) unilateral isometric 90° knee extension maximal voluntary torque (MVT) using an isokinetic dynamometer (Cybex Humac Norm Isokinetic Extremity System; Computer Sports Medicine Inc., Stoughton, MA, USA); and (7) 6-minute walk test (6MWT). Thereafter, patients were randomly allocated to groups as described above. At Visit 2, at least 48 hours after Visit 1, patients completed 1RM testing and arterial occlusion pressure as appropriate. Visits 3 to 18 were sessions of the supervised exercise program. One week following the supervised exercise program on Visit 19, measurements for vastus lateralis muscle thickness, ABI, QoL, MVT, and 6MWT were repeated.

### Feasibility and acceptability outcomes

The primary outcomes for this study were feasibility and acceptability of procedures for recruitment, allocation, measurement, and retention. Recruitment rates were measured as rate of invited patients who were eligible and consenting. Attrition rates were established as discontinuation of the intervention and loss to follow up. Reasons for drop-out were used to assess the suitability of allocation. Adherence was monitored by session attendance. Completion rates were defined as the number of patients attending the follow-up assessments.

The safety of the intervention was assessed by exploring reasons for dropout, and the number, type, and severity of adverse events that occur in each group. Patient safety was overseen by a comprehensive research team, including a study physician (SN).

The acceptability of procedures was assessed by using session adherence data and cardiovascular and perceptual responses describing patients’ exercise tolerance to the exercise sessions. Heart rate (HR) was monitored (TICKR; Wahoo, Atlanta, GA, USA) throughout exercise and blood pressure was assessed (HEM-8712; Omron Healthcare, Kyoto, Japan) immediately at the end of each exercise set. Patients’ perception of exercise intensity, exercise-induced pain, and affective valence was assessed immediately at the end of each exercise set using RPE,^36^ ratings of pain,^40^ and the feeling scale,^41^ respectively. Visual analogue scales (VAS, 0–10 cm) were used 10 minutes postexercise to assess patients’ perceived level of enjoyment, difficulty, fatigue, tolerance, effectiveness, and safety to the exercise session. A negative response was represented at 0 cm of the scale (e.g., not at all enjoyable) and positive response was represented at 10 cm of the scale (e.g., I enjoyed it very much). Measures of exercise tolerance were recorded on sessions 1, 8, and 16. The mean of the measures over the three sessions were used for analysis.

### Data analysis

Success criteria for the feasibility trial include: (1) obtaining sufficient 6MWT data to allow for a formal sample size calculation based on the SD of this specific dependant variable; (2) attendance of ⩾ 75% of scheduled sessions; (3) loss to follow up is < 20%; (4) there are no serious adverse events resulting from the trial procedures; and (5) there are no significant difficulties for the researcher in administering the procedures or the intervention, measured by missing outcome data. The success criteria of this trial provided the basis of interpretation to determine whether a definitive trial is feasible.

For measurements, continuous variables were described as mean ± SDs and frequency counts and percentages were provided for categorical data. As this study was not intended or powered to detect statistical differences in outcomes (e.g., *p*-value < 0.05), estimated mean differences with 95% CIs were presented where appropriate. All analyses were conducted using IBM SPSS, Version 26 (Armonk, NY, USA).

## Results

**Figure 3.**
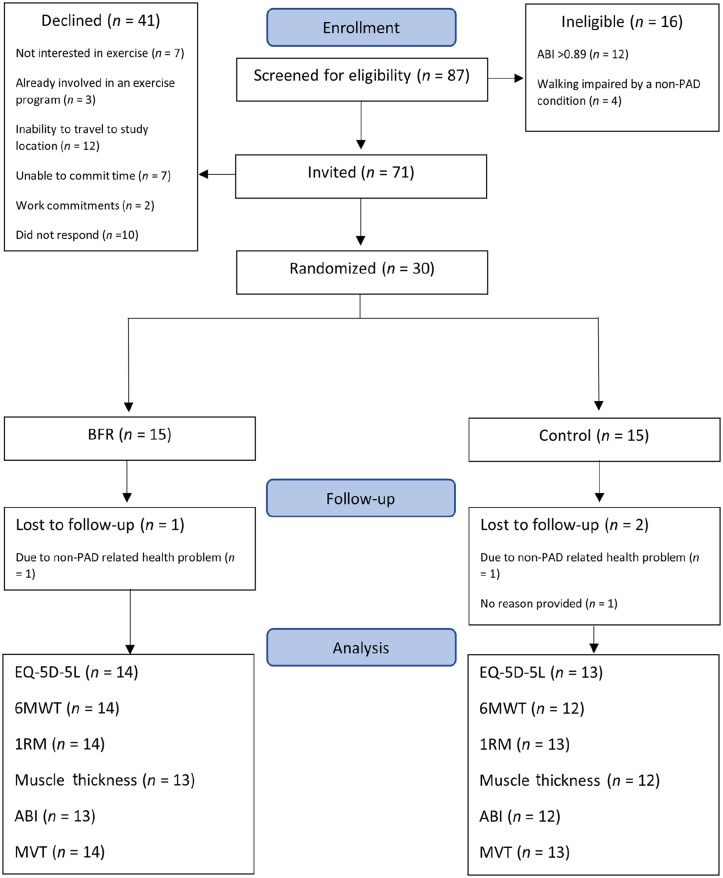
CONSORT flow diagram. ABI, ankle–brachial index; PAD, peripheral artery disease; BFR, blood flow restriction group; EQ-5D-5L, quality of life questionnaire; 6MWT, 6-minute walk test; 1RM, one-repetition maximum; MVT, maximal voluntary torque.

Figure 3 shows the flow of patients through the trial. Recruitment took place between April 2021 and March 2022 with all follow-up data collection completed by July 2022.

### Screening, eligibility, and recruitment

A summary of feasibility and acceptability data is presented in Table 1. All success criteria were met (e.g., there were no serious adverse events resulting from the trial procedures, 81% of exercise sessions were attended by patients, and the retention rate was 90%). Of 87 patients screened, 71 met the inclusion criteria, and 30 (24 men and six women) were recruited, giving eligibility and recruitment rates of 82% and 42%, respectively. Reasons for exclusion and declined participation are shown in Figure 3.

**Table 1.** Summary of trial feasibility and acceptability data.

Methodological issues	Findings	Evidence
What factors influenced eligibility and what proportion of those screened were eligible?	Sheffield Vascular Institute run a claudication clinic from which many patients referred would be eligible.	71/87 (82%) screened were eligible. All ineligible patients had an ABI indicative of non-PAD.
Was recruitment successful?	Yes	The target sample was achieved within a 12-month period.
Were eligible patients recruited?	Conversion to recruitment was sufficient to meet target.	12/41 (29%) eligible patients were recruited in the study. However, most patients who declined participation were unable to travel to the central study location (24%) and this could be improved by a change in study design in a future trial.
Were patients successfully randomized and did randomization yield equality in groups?	The randomization process worked well.	Equal-sized groups, well balanced on stratification and most other variables; however, 1RM was higher at baseline in the BFR group.
Did patients adhere to the exercise program?	Adherence was high for both groups.	Adherence to the exercise programs was 81% in total (BFR = 78%, control = 84%).
Was the exercise program acceptable to the patients?	Quantitative data suggest the exercise programs were acceptable.	There was no patient dropout due to allocation preference. VAS for enjoyment, difficulty, fatigue, tolerance, effectiveness, and safety were all rated positively for both groups.
Was the intervention safe?	Our safety data appear favorable.	No nonserious or serious adverse events were recorded during the study period.
Were outcome assessments completed?	Outcome completion rates were very high.	Completion rates was 90% in total (BFR = 93%, control = 87%).
Was sufficient 6MWT data obtained to allow for a formal sample size calculation?	Yes	Across both groups, 26 6MWT observations were obtained, which is sufficient for a formal power calculation for a definitive trial.
Was retention to the study good?	Retention was very high.	Retention rate was 90%.
Did all components of the protocol work together?	Yes	There were no major difficulties identified in the various processes and the researchers’ ability to implement them.
Was an appropriate outcome defined for the definitive trial?	Yes	The 6MWT and EQ-5D-5L questionnaire appear to be the most appropriate outcomes for a definitive trial.

ABI, ankle–brachial index; BFR, blood flow restriction group; PAD, peripheral artery disease; VAS, visual analogue scale; 1RM, one-repetition maximum; 6MWT, 6-minute walk test.

### Patient characteristics

Patients’ characteristics were similar between the two groups at baseline, except for age (Table 2). The groups were well balanced at baseline for most variables (Table 3).

**Table 2.** Patients’ baseline characteristics.

	BFR (*n* = 15)	Control (*n* = 15)
Age, years	66.8 ± 8.6	71.6 ± 9.1
Female sex	3 (20%)	3 (20%)
Race, White	100%	100%
Height, cm	168.6 ± 11.7	171.1 ± 10.2
Body mass, kg	79.4 ± 12.3	77.6 ± 12.1
BMI, kg·m^2^	27.9 ± 3.1	26.5 ± 3.5
ABI	0.62 ± 0.16	0.69 ± 0.11
Bilateral claudication	7 (47%)	5 (33%)
COPD	3 (10%)	2 (7%)
Ischemic heart disease	6 (20%)	5 (17%)
Current smoker	4 (27%)	3 (20%)
Previous smoker	14 (93%)	11 (73%)
Medications		
Antiplatelet agent	15 (100%)	15 (100%)
Statin	15 (100%)	15 (100%)

Data are mean ± SD or *n* (%).

a Independent *t*-test; ^b^chi-squared test.

ABI, ankle–brachial index; BFR, blood flow restriction; BMI, body mass index; COPD, chronic obstructive pulmonary disease.

**Table 3.** Change in physical function assessments and muscle thickness.

Variable	Baseline	Follow up	% Difference
**6MWT**			
Distance (m)			
BFR	371.3 ± 91.9	426.5 ± 102.2	15%
Control	372.4 ± 98.4	408.7 ± 104.1	10%
Time to claudication (s)			
BFR	127.5 ± 68.5	172.2 ± 59.8	35%
Control	113.6 ± 65.5	111.0 ± 55.0	−2%
Pain (CR-10^+^)			
BFR	4.3 ± 2.6	3.1 ± 1.8	−28%
Control	4.3 ± 1.4	4.5 ± 2.2	6%
**Muscle thickness (mm)**			
BFR	21.9 ± 2.7	21.8 ± 3.3	0%
Control	21.7 ± 4.1	22.1 ± 4.2	2%
**ABI**			
BFR	0.64 ± 0.15	0.67 ± 0.21	5%
Control	0.74 ± 0.11	0.72 ± 0.14	−3%
**MVT**			
BFR	126.3 ± 37.1	123.5 ± 38.3	−2%
Control	105.3 ± 50.1	102.5 ± 47.2	−3%

Data are mean ± SD.

ABI, ankle–brachial index; BFR, blood flow restriction; CR, category-ratio; MVT, maximal voluntary torque; 6MWT, 6-minute walk test.

### Retention, adherence, and completion

The retention rate was 90%. One patient from each group formally left the study due to a non-PAD-related health issue. Another patient in the control group left the study but did not provide a reason. Adherence to the exercise programs was 81% in total (BFR = 78%, control = 84%). The completion rate was 90% in total (BFR = 93%, control = 87%).

### Safety and exercise tolerance

No adverse events or serious adverse events were recorded during the study period. Cardiovascular and perceptual responses describing patients’ exercise tolerance are presented in Table 4.

**Table 4.** Outcomes for exercise tolerance.

	BFR	Control
HR_peak_ (bpm)	101 ± 12	94 ± 27
SBP_peak_ (mmHg)	180 ± 27	170 ± 34
DBP_peak_ (mmHg)	100 ± 14	93 ± 16
RPE (CR-20)	14.1 ± 1.8	12.9 ± 1.7
	(‘somewhat hard’ to ‘hard’)	(‘somewhat hard’)
Pain (CR-10^+^)	4.2 ± 2.0	3.4 ± 1.6
	(‘moderate’ to ‘strong’)	(‘moderate’)
Feeling scale (–5/+5)	3.5 ± 1.9	3.9 ± 1.0
	(‘good’)	(‘good’)
Visual analogue scales (0–10 cm)		
Enjoyment	8.9 ± 1.2	9.4 ± 0.6
Difficulty	3.7 ± 2.3	3.9 ± 3.0
Fatigue	2.3 ± 2.0	3.3 ± 3.0
Tolerance	7.9 ± 1.8	8.3 ± 1.5
Effectiveness	8.7 ± 0.8	8.9 ± 0.7
Safety	9.7 ± 0.3	9.8 ± 0.2

Data are mean ± SD.

BFR, blood flow restriction; CR, category-ratio; DBP, diastolic blood pressure; HR, heart rate; RPE, ratings of perceived exertion; SBP, systolic blood pressure.

### Outcome measurements

**Figure 4.**
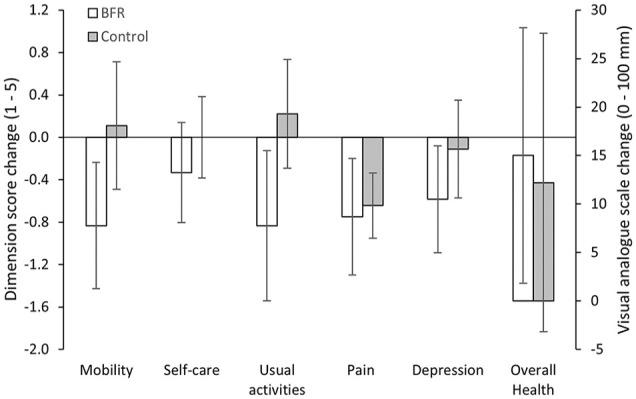
Change in quality of life assessed using the EQ-5D-5L questionnaire at baseline and follow up. Data are mean difference with 95% CIs. BFR, blood flow restriction.

Data from physical function assessments and muscle thickness are presented in Table 3. Both groups observed an overall improvement in 6MWT distance (BFR = 55.2 m [42.4, 67.9], control = 36.3 m [10.8, 61.8]). However, at an individual level, 86% of patients in BFR improved their 6MWT distance by > 35.5 m (which represents a large clinically important difference) at follow up compared with 33% of patients in the control. Additionally, time to claudication during 6MWT was prolonged at follow up for BFR (44.7 s [20.8, 68.6]) but not the control (2.6 s [−23.2, 28.4]), and ratings of pain at the end of the 6MWT may have been reduced for BFR (1.1 CR-10^+^ [−0.1, 2.4]) but not the control (–0.3 CR-10^+^ [−1.4, 0.8]). Vastus lateralis muscle thickness, ABI, and MVT did not change at follow up for either group. Change in QoL assessed using the EQ-5D-5L questionnaire is presented in Figure 4. Patients’ QoL was similar between the two groups at baseline. QoL improved for BFR, with score reductions in four out of five dimensions and increased self-rated overall health, but did not improve for the control, with only one dimension score reduction.

## Discussion

The aim of the study was to evaluate the feasibility of a supervised BFR program in patients with claudication. The primary finding was that BFR was feasible in patients with claudication with all success criteria being met. Additionally, it was shown that BFR has the potential to increase exercise performance, reduce pain, and improve QoL. Our findings support the progression to a definitive, multicenter trial to evaluate the clinical and cost-effectiveness of BFR in patients with claudication.

A key element of the success of the trial was patient recruitment. The target number of patients was achieved within the trial timeframe despite the COVID-19 pandemic imposing substantial pressure on clinical services. A strong commitment by the clinical team to approach, promote, and invite was the driving force of recruitment, alongside a flexible and friendly approach from the research team, which will be a decisive factor for the success of a definitive trial. Another recruitment success component was contributed to by the Sheffield Vascular Institute delivering claudication-specific clinics. This likely increased the frequency of screening patients who fit the inclusion criteria, which is evident by the high eligibility rates observed in the study.

Despite recruitment rates in the study being acceptable, they could be improved. From eligible patients who were invited to the study but declined, 29% of the responses were due to inability to travel to the study location. The difficulty/inability to travel to central exercise locations from the city outskirts because of time, cost, or accessibility poses a significant barrier to participation for many patients.^42^ Delivery of sessions with an option of times in community-based venues increases accessibility for patients and may improve inclusivity and uptake for a future trial.^43^

High adherence rates for BFR are an encouraging sign of the acceptability of this exercise method in patients with claudication, which was supported by the implementation of the six pillars of adherence framework developed by our team.^42^ To support this, the average feeling scale response during exercise was ‘good’, and VAS reports following exercise were positive for perceived enjoyment, tolerance, difficulty, effectiveness, and safety. Furthermore, no adverse or serious adverse events were reported during the study that were attributed to the study procedures.

A contributing factor to high adherence could be attributed to the frequency of sessions and program duration employed in the study, which is relatively low compared to traditional exercise prescription for this patient group of three times a week for 12–24 weeks.^44^ It has been observed that studies which report the lowest levels of adherence tend to be those with higher exercise frequencies and longer durations.^4^ Although the high adherence rates in this study is a good achievement, a future trial should incorporate cognitive-behavioral strategies to optimize exercise adherence and encourage a lasting change in behavior and lifestyle to further patient benefit.^45^

The outcomes of the 6MWT in the present study hold potential for explaining in a fully powered randomized trial the extent to which BFR could affect walking performance in patients with claudication. The 6MWT is a well-validated measure of walking performance which reflects normal walking and requires minimal resources.^46^ The 6MWT has excellent intraday test-retest reliability in patients with claudication,^47^ improves in response to exercise interventions,^48,49^ and predicts rates of mobility loss and mortality.^50^ Additionally, the minimally clinically important difference in 6MWT has been defined in patients with claudication.^51^

Though the data in the present study should not be over-interpreted, it is encouraging to observe a 15% group increase in 6MWT distance, of which 86% of patients increased their 6MWT distance by > 35.5 m, which represents a large clinically important difference,^51^ and time to claudication and pain were improved. An improved tolerance to exercise and pain can have large implications in the QoL of patients with claudication.^52,53^ The current study’s findings indicate an improved QoL with mobility, ability to do usual activities, pain, depression, and overall health rated more positively at follow up.

BFR augmented cardiovascular responses, exertion, and pain, which may be indicative of a stimulus that improves cardio-respiratory physiology and muscle conditioning that can increase exercise performance.^54^ Additionally, higher pain experienced during BFR may have habituated patients to the pain level contributing to increased time to claudication and lower pain ratings during the 6MWT. Exercise is known to decrease sensitivity to pain, and low-intensity exercise performed with BFR has been shown to induce hypoalgesia.^55^ Further research is required to explain the mechanisms which BFR improves exercise performance in patients with claudication. Interestingly, no changes were observed for vastus lateralis muscle thickness and MVT. This was unexpected given that studies frequently demonstrate hypertrophy and strength adaptation following BFR.^17^ To observe changes in these outcomes, increased exercise load (~30% 1RM), frequency, or program duration may be required.

Supervised resistance exercise programs have repeatedly been shown to improve claudication onset distance and total walking distance in patients with claudication.^48,56,57^ Studies report greater improvements in walking performance when high-intensity (⩾ 70% 1RM) resistance exercise is performed.^58^ Importantly, a supervised program of low-intensity (20–30% 1RM) resistance exercise resulted in no change in walking performance in patients with claudication.^56^ The findings in the present study are promising, as they demonstrate the potential of greater changes to walking performance when BFR is applied with low-intensity resistance exercise. This has potential clinical relevance as low-intensity resistance exercise with BFR may be beneficial to patients where high-intensity resistance exercise is contraindicated or unfeasible. This highlights the need for a future study to make comparisons between low-intensity resistance exercise with BFR and high-intensity resistance exercise, to further understand the impact of BFR.

This study has presented a novel exercise mode which has potential for improving exercise performance, QoL, and exertional leg symptoms in patients with claudication. Conducting this feasibility trial has allowed procedures and protocols to be tested. Modelling an intervention before a full-scale evaluation can identify weaknesses, lead to refinement, and indicate whether a full-scale trial is warranted.^30^ The outcome of this trial has reduced uncertainty around recruitment, retention, measurements, and the proposed intervention and has provided recommendations to refine design to improve content and delivery of intervention, acceptability, and adherence.

### Study limitations

The study is not without limitations. Lack of women and ethnic diversity in the sample limits the generalizability of the findings. The majority (86%) of patients in the study were White men. Women and ethnic minorities are often underrepresented in PAD prospective randomized controlled trials^59^ and more efforts need to be made to increase the participation of women and ethnic groups to obtain a sample that is representative of the PAD population. This was not possible due to the limited trial funding, which had a knock-on effect on staff resources. Additionally, a future trial should consider integrating qualitative methods to provide a more comprehensive evaluation beyond effectiveness. Furthermore, the study did not have a standard control group consisting of patients receiving usual care. The inclusion of a usual-care group as a third arm in the study has potential to offer more rigorous evidence to support the interpretation of the feasibility of BFR and should be considered in a future clinical and cost-effectiveness trial to provide the most clinical value.

The priority for future research on this topic is the progression to a full-scale definitive trial. Such a trial should engage stakeholders, namely patients and NHS collaborators, from the outset. Meaningful engagement with stakeholders at each stage of the research will maximize the potential of developing the intervention to be more effective for real-world applications.^30^ A future trial should consider whether it is accessible to patients from disadvantaged socioeconomic groups. This may require active and targeted recruitment, engagement with community stakeholders and organizations, and ensuring research personnel are well trained to match the population of interest to encourage accessibility and appealability to these groups.^60^ Economic considerations should be a component of any future trial. Amending the research design to group sessions from one-to-one sessions, thereby increasing the supervisor to patient ratio, will be more efficient and improve the cost effectiveness of the intervention. Additionally, group sessions offer social support to patients, which is an important element for intervention design.^5^ Incorporating cognitive-behavioral strategies within the study can encourage positive behavior and lifestyle change which would benefit patients beyond the exercise program. Lastly, a future trial should examine the potential mechanisms by which BFR improves exercise performance and exertional leg symptoms. These findings should be compared against the gold standard exercise therapy, walking exercise, and traditional resistance exercise programs (60–80% 1RM) to determine whether BFR provides patients with additional benefit.

## Conclusion

Our findings support the feasibility and acceptability of a supervised BFR program in patients with claudication, observing good recruitment rates, low attrition rates, high adherence rates, and no adverse events. In addition, our results suggest that 8 weeks of blood flow restriction has the potential to increase exercise performance, reduce pain, and improve QoL. The next step will be the design and implementation of an appropriately powered, multicenter trial, which is required to assess the clinical and cost-effectiveness of the intervention.
